# A rare case of multiple myeloma with double translocations: t(11;14) and t(14;16)

**DOI:** 10.1007/s12308-025-00626-w

**Published:** 2025-03-27

**Authors:** Mina Meseha, Vishw Patel, Kirolos Sekla, Ping Yang, David Coffey

**Affiliations:** 1 University of Miami, Miami, USA; 2 Cleveland Clinic, Cleveland, USA; 3 University of Alexandria, Alexandria, Egypt

**Keywords:** Multiple myeloma, Double translocation myeloma, t(11;14), t(14;16), Cytogenetics, Chromosomes

## Abstract

Multiple myeloma is a clonal plasma cell malignancy often characterized by complex cytogenetic abnormalities that influence prognosis and treatment strategies. This report describes a 63-year-old male with kappa light chain multiple myeloma and a rare finding of double translocation involving t(11;14) and t(14;16), detected by FISH analysis. This case emphasizes the clinical implications of such genetic abnormalities and their impact on disease progression and therapeutic decisions.

## Introduction

Multiple myeloma (MM) is a plasma cell neoplasm characterized by a wide array of genetic mutations and translocations that significantly influence its pathogenesis and clinical outcomes. The concept of “double-hit” myeloma refers to a genomically defined high-risk subgroup of patients characterized by an exceptionally unfavorable prognosis, even when treated with advanced therapies (with estimated 18-month progression-free survival and overall survival rates of 39% and 48%, respectively). A comprehensive genomic analysis is required to detect two specific genetic abnormalities: bi-allelic alterations in *TP53* and gain/amplification of *1q* [1].

These markers help to predict prognosis, yet treatment approaches remain uniform [2–4]. Conversely, “double translocation” does not imply “double-hit status.”

In this case report, we present a rare instance of double translocation involving t(11;14) and t(14;16) in a patient with relapsed MM. The t(11;14) translocation leads to the overexpression of Cyclin D1 and inactivation of the tumor suppressor gene *RB1*, facilitating uncontrolled cell cycle progression. Concurrently, the t(14;16) translocation results in the overexpression of the transcription factor c-MAF, which subsequently upregulates Cyclin D2 and promotes increased DNA synthesis and cell proliferation. While the prognostic significance of t(11;14) MM remains debated [5, 6], studies have shown that patients with t(11;14) or high *BCL2* gene expression experience improved responses and progression-free survival [7, 8]. In contrast, t(14;16) is highly aggressive and is associated with a poor prognosis, particularly when combined with other high-risk abnormalities [9, 10].

The dual involvement of the immunoglobulin heavy chain (*IGH*) gene in two distinct chromosomal translocations is exceptionally rare and highlights the intricate nature of MM biology.

## Case report

A 63-year-old male with no significant past medical history initially presented with complaints of chest pain and back pain. Laboratory results revealed hemoglobin at 8.6 g/dL, serum calcium at 11.4 mg/dL, serum creatinine at 1.4 mg/dL, low quantitative immunoglobulins, albumin at 4.5 g/dL, and B2-microglobulin at 14.1 mg/dL. Serum protein electrophoresis revealed a 0.1 g/dL monoclonal protein band in the gamma region, and serum immunofixation was positive for the free kappa light chain (FKLC). His FKLC was 4190 mg/dL with a kappa/lambda ratio of 17,000. Subsequent positron emission tomography/computed tomography (PET/CT) imaging revealed diffuse lytic lesions in the right lateral ribs, left lateral sixth rib, T12, right iliac, and proximal femur.

A bone marrow biopsy was obtained and showed plasma cells comprising 90% of overall marrow cellularity by CD138 immunohistochemistry. Plasma cells were enriched from bone marrow aspirate using CD138 MicroBeads (Miltenyi Biotec, Gaithersburg, MD, USA). Interphase fluorescence in situ hybridization (FISH) was performed on the enriched plasma cells according to the manufacturer’s protocols. Probes included Vysis LSI TP53/D17Z1, CDKN2C/CKS1B, D13S319/LAMP1, CEP9, and CEP15, as well as dual-color, dual-fusion probe sets for FGFR3/IGH, CCND1/IGH, IGH/MAF (Abbott Molecular Diagnostics, Illinois, USA), and IGH/*MAFB* (MetaSystems, Medford, MA, USA). For each probe, 100 cells were manually scored by two independent technologists using microscopy. FISH analysis detected a 13q deletion in 98% of the cells, and an *CCND1-*IGH fusion t(11;14) in 94% of the cells. Other probes used, including D7S486/CEP7 (7q31), FGFR3/IGH, IGH/MAF, TP53 (17p13)/CEP17, CEP3, CEP9, and CEP15, all tested normal (Table 1). He was diagnosed with kappa light chain multiple myeloma, International Staging System stage III.

**Table 1:** Myeloma-specific FISH findings in the bone marrow biopsy at diagnosis and new double translocation finding

Probes	Screen	BMBx at diagnosis		BMBx with double translocation	
		**% abnormal cells**	**Abnormality**	**% abnormal cells**	**Abnormality**
**D7S486/CEP7 (7q31)**	**Chromosome 7 abnormality**	0	None	Not tested	n/a
**IGH (14q32)**	**IGH gene**	94	1 copy 5’ IGH sep 3’ IGH	Not tested	n/a
**IGH/CCND1**	**t (11;14)**	93	**t (11;14)** 1–2 fusions/2–3 CCND1/1–3 IGH	74	**t (11;14)** 2–4 fusions/2–7 copies CCND1/2–4 copies IGH
**FGFR3/IGH**	**t (4;14)**	30	2 copies FGFR3/1 copy IGH	75	3 copies FGFR/3–4 copes IGH
**IGH/MAF**	**t (14;16)**	27	1 copy IGH/2 copies MAF	89	**t (14;16)** 3–4 fusions/3–4 copies IGH/3–8 copies MAF
**IGH/MAFB**	**t (14;20)**	Not tested	n/a	83	3–6 copies MAFB/3–4 copies IGH
**TP53 (17p13)/CEP17**	**17p del**	0	None	70.5	4 copies TP53/3–4 copies 13q34
**D13S319/LAMP1**	**13q del**	98	D13S319 × 1/LAMP1 × 2	72/12	2 copies 13q14/3–4 copies 13q34
**CEP3**	**3 gain**	0	None	Not tested	n/a
**CEP9**	**9 gain**	0	None	56/24	9 gain (trisomy, tetrasomy)
**CEP15**	**15 gain**	0	None	28/44	15 gain (trisomy, tetrasomy))
**CDKN2C/CKS1B**	**1p/1q del/gain, amp**	Not tested	n/a	84	3 copies 1p/3–4 copies 1q

## Clinical course and treatment

The patient started on bortezomib–lenalidomide–dexamethasone. Post-induction bone marrow biopsy revealed 5% plasma cells, with FISH positive for *CCND1-IGH* fusion in 18% of cells and 13q deletion positive in 10% of cells. Following this, the patient underwent autologous stem cell transplant and transitioned to lenalidomide maintenance.

He remained in remission for 1 year before experiencing multiple relapses. The first relapse occurred when he developed neck pain due to a lytic lesion and pathologic fracture of C2. A repeat bone marrow biopsy showed no evidence of disease; however, due to rising FKLC and the new lytic lesion, he was started on daratumumab–pomalidomide–dexamethasone.

The second relapse occurred 1 year later, characterized by rising FKLC and a repeat bone marrow biopsy showed 35% plasma cells by CD138 immunohistochemistry on core biopsy. FISH analysis was not reported due to the limited sample. Consequently, his therapy was changed to carfilzomib–pomalidomide–dexamethasone (KPd).

Despite this treatment, his FKLC level continued to rise while receiving KPd. Another bone marrow biopsy revealed 35% plasma cells, with FISH detecting IGH rearrangement in 8% of cells and *CCND1-IGH* fusion in 9% of cells; the prior 13q deletion was not detected (Table 2). This led to enrollment in a clinical trial to receive linvoseltamab, a BCMA/CD3 bispecific antibody (NCT03761108).

**Table 2:** FISH results at key time points

Period	Plasma cells %	FISH
At diagnosis	90	*CCND1-IGH* fusion t(11;14) in 94% of the cells and 13q deletion in 98% of cells
Post-induction	5	*CCND1-IGH* in 18% of cells and 13q deletion positive in 10% of cells
First relapse	No PCs	n/a
Second relapse	35	not reported due to the limited sample
Third relapse	35	*CCND1-IGH* in 9% of cells; the prior 13q deletion was not detected
Fourth relapse	No PCs	n/a
Fifth relapse	5	*CCND1/IGH* and *IGH/MAF* were detected in 74% and 89% of cells

**Fig. 1:**
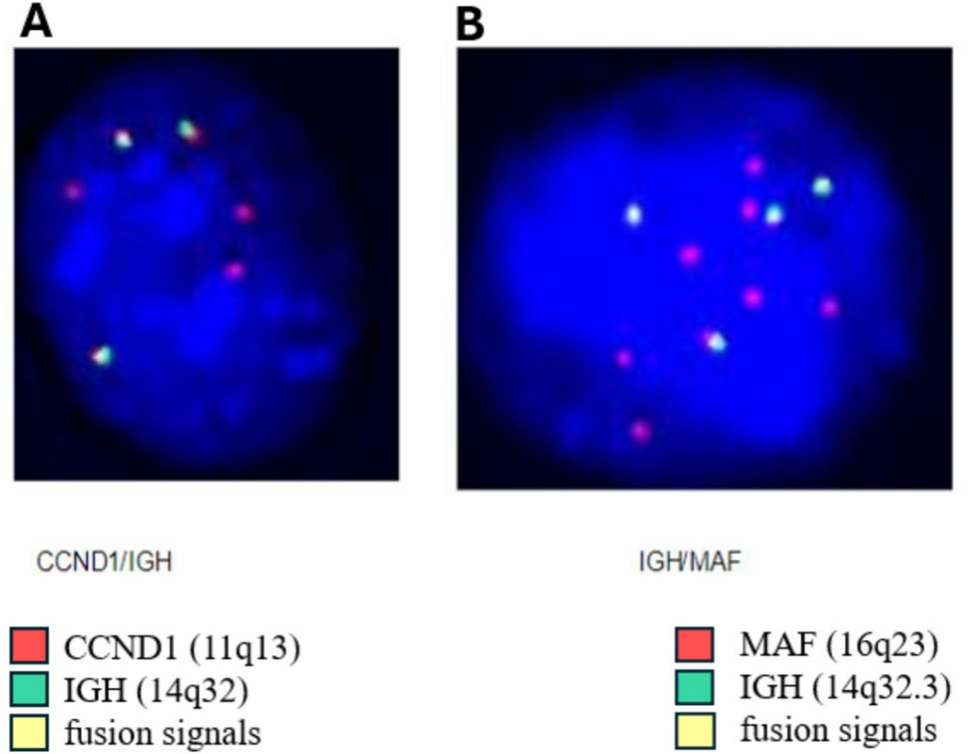
Fluorescence in situ hybridization (FISH) on plasma cell nucleus showing double translocations of t(11;14) and t(14;16). **A** FISH with probes revealing t(11;14); *CCND1* at 11q13 fused to *IGH* gene at 14q32 resulting in fusion signals. **B** FISH with probes revealing t(14;16); *IGH* gene region at 14q32.3 fused *MAF* gene region at 16q23 resulting in fusion signals

Eighteen months later, a PET/CT showed new FDG-avid osseous lesions in the left greater trochanter and left ischium. A biopsy of the latter confirmed plasma cell neoplasm, although a bone marrow biopsy showed no evidence of plasma cell neoplasm. As a result, he was taken off the bispecific antibody therapy and started on carfilzomib–venetoclax–dexamethasone. A repeat PET/CT after ten months demonstrated an increase in the number and metabolic activity of multifocal FDG-avid osseous lesions. A subsequent bone marrow, flow cytometry analysis showed 5% plasma cells with abnormal plasma cells comprising 84% of plasma cells and 2.67% of leukocytes.(Figure 1)

FISH analysis revealed patterns indicating 1–2 extra copies of 1p, 1q, *FGFR3*, CEP9, 13q34, CEP17, and *TP53* in 24 to 84% of cells examined, suggesting the presence of partial or whole trisomy or tetrasomy of chromosomes 1, 4, 9, 13, and 17. Additionally, atypical 2–4 fusions of *CCND1/IGH* and 3–6 fusions of *IGH/MAF* were detected in 74% and 89% of cells, respectively. Extra copies of genes were also observed, with 1–3 extra copies of *CCND1* in 74% of cells, 1–4 extra copies of *MAF* in 89% of cells, and 1–4 extra copies of *MAFB* in 83.5% of cells, indicating partial or whole extra copies of chromosomes 11, 16, and 20. The patient was enrolled in a clinical trial (NCT05308654) investigating a next-generation BCL-2 inhibitor, ABBV-453, for 2 months. However, he experienced a relapse and transitioned to ixazomib–cytoxan–dexamethasone as a bridge to CAR-T cell therapy. Unfortunately, he passed away after six years of battling the disease.

## Discussion

MM is a highly heterogeneous disease characterized by diverse genetic abnormalities, clinical presentations, and treatment responses [11, 12]. The disease is driven by primary events, such as hyperdiploidy and IGH locus translocations, as well as secondary events, including 1q21 gains/amplifications, 17p deletions, and *MYC* gene translocations [10]. The most frequent IGH translocations—t(11;14), t(4;14), and t(14;16)—occur in approximately 15–20%, 10–25%, and 3–7% of newly diagnosed MM patients, respectively [13]. Double translocations in MM, such as the co-occurrence of t(11;14) and t(14;16) observed in our case, are exceedingly rare. To our knowledge, this is the first reported case of t(11;14) and t(14;16) occurring together. Only one prior case report has described double translocations involving IGH, specifically t(4;14) (*FGFR3/IGH* fusion) and t(14;16) (*IGH/MAF* fusion), while eight other cases have documented double translocation myeloma with both t(8;14) (*IGH/MYC*) and t(11;14) (*IGH/CCND1*) translocations [1, 14–16].

In our case, all IGH loci involved in both *CCND1* and *MAF* fusions showed no normal IGH locus (green color) detected by FISH, suggesting that the translocations originated from the same IGH loci. The IGH enhancer likely drives the high expression of both *CCND1* and *MAF*. However, advanced molecular techniques such as optical genome mapping and DNA sequencing are needed to better understand the detailed molecular structural rearrangements underlying these double translocations.

The genetic landscape of MM can evolve over time, leading to the emergence of new chromosomal abnormalities or translocations such as t(14;16), which was not initially detected in our case. This phenomenon can be attributed to genomic instability and clonal evolution of MM, where the disease comprises multiple subclones of myeloma cells, each with distinct genetic profiles. As the disease progresses, selective pressures from the microenvironment or treatments can lead to the expansion of subclones and the acquisition of new genetic abnormalities [17, 18].

Moreover, the sensitivity of detection techniques, such as FISH and next-generation sequencing, plays a critical role. These methods may fail to detect minor subclones with specific genetic abnormalities if those subclones are present at very low levels initially but become detectable as they expand under selective pressures [19]. Furthermore, exposure to high-dose chemotherapy (e.g., melphalan) drives mutations during relapse. These mutational processes contribute significantly to the genetic complexity of MM, influencing disease progression and response to treatment [20–22].

Our patient’s therapeutic journey, involving multiple lines of treatment and clinical trials, highlights the challenges of managing relapsed/refractory MM with complex genetic profiles. We propose that the emergence of the t(14;16) translocation following treatment with a BCL-2 inhibitor may have contributed to disease progression by creating a selective advantage for a pre-existing t(14;16) subclone to expand or by driving its de novo emergence. In patients with t(11;14) translocation, there is overexpression of BCL-2 and usually low expression of MCL-1 and BCL-XL proteins [23, 24]. Hence, medication like venetoclax, which is a BCL-2 inhibitor**,** has proven to be an effective treatment [25]. The emergence of a t(14;16) translocation in a patient with a t(11;14) translocation could significantly affect the therapeutic efficacy of this medication [26]. The t(14;16) translocation leads to increased expression of anti-apoptotic proteins BCL-XL and MCL-1 [27]. This shift in the expression of anti-apoptotic proteins may have made this patient refractory to venetoclax [23, 27].

## Conclusion

This case report illustrates the clinical course of kappa light chain multiple myeloma patient characterized by the rare presence of double translocations t(11;14) and t(14;16) detected via FISH analysis. The patient’s disease progression was marked by multiple relapses and complex genetic abnormalities. This case emphasizes the critical role of advanced genetic analysis in understanding multiple myeloma’s heterogeneous nature and in formulating individualized treatment strategies moving forward.

## Data Availability

No datasets were generated or analysed during the current study.
